# Podocyte-targeted Heme Oxygenase (HO)-1 overexpression exacerbates age-related pathology in the rat kidney

**DOI:** 10.1038/s41598-020-62016-9

**Published:** 2020-03-31

**Authors:** Elpida Poulaki, Maria G. Detsika, Eythimia Fourtziala, Elias A. Lianos, Hariklia Gakiopoulou

**Affiliations:** 1 First Department of Pathology, School of Medicine, National and Kapodistrian University of Athens, 75 Mikras Asias Street, Athens, 11527 Greece; 2 First Department of Critical Care Medicine & Pulmonary Services, Evangelismos Hospital, National and Kapodistrian University School of Medicine, 3 Ploutarchou Street, Athens, 10675 Greece; 3 Veterans Affairs Medical Center and Virginia Tech. Carilion School of Medicine, 1970 Roanoke Blvd, Salem, VA 24153 USA

**Keywords:** DNA damage response, Chronic kidney disease

## Abstract

Although Heme Oxygenase-1 (HO-1) induction in various forms of kidney injury is protective, its role in age-related renal pathology is unknown. In the ageing kidney there is nephron loss and lesions of focal glomerulosclerosis, interstitial fibrosis, tubular atrophy and arteriolosclerosis. Underlying mechanisms include podocyte (visceral glomerular epithelial cell/GEC) injury. To assess whether HO-1 can attenuate ageing – related lesions, rats with GEC-targeted HO-1 overexpression (GEC^HO-1^ rats) were generated using a Sleeping Beauty (SB) transposon system and extent of lesions over a 12-month period were assessed and compared to those in age-matched wild-type (WT) controls. GEC^HO-1^ rats older than 6 months developed albuminuria that was detectable at 6 months and became significantly higher compared to age-matched WT controls at 12 months. In GEC^HO-1^ rats, lesions of focal segmental and global glomerulosclerosis as well as tubulointerstitial lesions were prominent while podocytes were edematous with areas of foot process effacement and glomerular basement membrane thickening and wrinkling. GEC^HO-1^ rats also developed hemoglobinuria and hemosiderinuria associated with marked tubular hemosiderin deposition and HO-1 induction, while there was depletion of splenic iron stores. Kidney injury was of sufficient magnitude to increase serum lactate dehydrogenase (LDH) and was oxidative in nature as shown by increased expression of 8-hydroxydeoxyguanosine (8-OHdg, a byproduct of oxidative DNA damage) in podocytes and tubular epithelial cells. These observations highlight a detrimental effect of podocyte-targeted HO-1 overexpression on ageing-related renal pathology and point to increased renal iron deposition as a putative underlying mechanism.

## Introduction

The ageing kidney is characterized by nephron loss and lesions of arteriolonephrosclerosis consisting of glomerular scarring, interstitial fibrosis and tubular atrophy. These lesions are associated with progressive deterioration of renal function and increased prevalence of end stage renal disease^1^. The ageing rat kidney has been extensively studied as a model to understand mechanisms underlying development of the aforementioned ageing-related lesions. Early studies by Bolton^2^ and Owen^3^ using the Sprague-Dawley rat described specific lesions developing in an age-dependent manner and consisting mostly of glomerular sclerosis, thickening of Bowman’s capsule, interstitial inflammatory infiltrates, interstitial fibrosis and tubular damage^2^.

Mechanisms underlying development of age-related glomerulosclerosis have recently been elucidated and emphasized the role of podocyte injury. It was demonstrated that podocyte injury is a prominent feature in ageing rat kidneys leading investigators to propose that the ageing kidney is primarily a podocyte senescence disorder^4,5^. Specifically, podocyte number decreases, and a subset undergo hypertrophy-related stress with reduction in nephrin levels^6^ and increase in those of desmin^4^. On the other hand, experimentally induced selective podocyte depletion results in glomerulosclerosis lesions analogous to those observed in the ageing glomerulus^7^. Moreover, in the aging rat kidney, there is increased iron accumulation and oxidative stress/injury^8^, which was shown to contribute to podocyte senescence^9^.

The inducible heme oxygenase isoform (HO-1) is upregulated by a variety of pathophysiologic factors causing oxidative stress and attenuates extent of cell injury^10,11^. The role of HO-1 in cell senescence varies depending on cell type. Aging reduces HO-1 levels in rat hepatocytes^12^ and in articular cartilages and menisci of mice^13^. Further, in cultured rat primary articular hondrocytes, HO-1 overexpression delays senescence^14^. In contrast, HO-1 is significantly overexpressed in neurons and astrocytes of ageing and of Alzheimer’s disease brains^15^ while in models of astroglial stress sustained Hmox1 induction promotes oxidative mitochondrial membrane damage, iron sequestration and mitophagy (macroautophagy)^16^.

The role of HO-1 in attenuating podocyte injury in the ageing-related kidney is unknown. Previous work on the protective role of HO-1 in rat podocyte injury consequent to oxidative stress resulting from systemic inhibition of Nitric Oxide (NO) production demonstrated that treatment with the HO-1 inducer, heme, preserved podocyte structural/functional integrity and ameliorated extent of glomerular and tubulointerstitial lesions^17^. However, although heme treatment increased total kidney HO activity, no HO-1 induction in podocytes was demonstrated indicating that renoprotective effects observed could be related to HO-1 induction in cells other than podocytes or to effects of heme other than HO-1 induction.

In contrast to other renal epithelial cells, podocyte HO-1 expression does not increase in response to exogenous administration of HO-1 inducers including heme^18^ or in response to various forms of glomerular injury in which potent HO-1 inducers, including cytokines and reactive oxygen and nitrogen radicals, are produced within glomeruli^19^. The question of whether HO-1 induction in podocytes attenuates ageing-related podocyte injury can, therefore, be addressed by achieving podocyte-targeted HO-1 overexpression. The present study addressed this question in ageing rats (2, 4, 6 and 12 months of age) with podocyte-targeted HO-1 overexpression generated using a hyperactive Sleeping Beauty (SB) transposon system.

## Results

### Podocyte-targeted HO-1 over expression exacerbates age-related albuminuria and glomerular lesions

**Figure 1:**
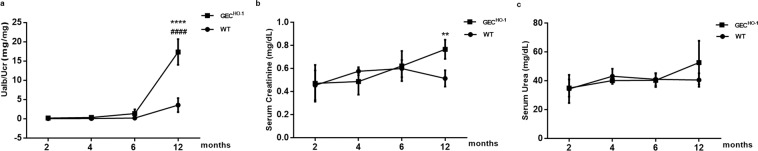
Changes in urine albumin excretion, serum creatinine and serum urea levels in GEC^HO-1^ and WT rats with time. Albuminuria, expressed as Ualb/Ucr (mg albumin/mg creatinine) (**a**), Serum Creatinine (mg/dL) (**b**) and Serum Urea (mg/dL) (**c**) in 2-, 4-, 6-, and 12-month old GEC^HO-1^ rats and WT controls. Values are expressed as mean ± SEM. ****p < 0.0001 12 month old GEC^HO-1^ vs 12 month old WT, ^####^p < 0.0001 12 month old GEC^HO-1^ vs 2 month old GEC^HO-1^, **p < 0,01 12 month old GEC^HO-1^ vs 12 month old WT.

Changes in urine albumin excretion (Ualbumin/Ucreatinine ratio, Ualb/Ucr) in GEC^HO-1^ and age-matched WT controls at 2, 4, 6, and 12 months of age are shown in Fig. 1a. Albuminuria was detected in 6-month old GEC^HO-1^ rats and became significantly higher compared to age-matched WT controls at 12 months. As albuminuria was observed following the 6-month time point we focused on the 2, 6 and 12-month timepoints for quantification of renal tissue injury. At 12 months, serum creatinine levels in GEC^HO-1^ rats were also significantly higher compared to age matched controls with no difference in serum urea levels (Fig. 1b,c). In addition to presence of albumin, urine analysis including sediment examination revealed presence of hemoglobin but absence of erythrocytes in centrifuged urine (hemoglobinuria) Table 1.

**Table 1:** Semi-qualitative assessment (described in methods) of hemoglobin presence in urine samples of 12-month old GEC^HO-1^ rats and WT age matched controls.

Urine Hemoglobin		
Rat	WT	GEC^HO-1^
#1	−	+++
#2	+	+++
#3	−	+++
#4	−	+++

**Figure 2:**
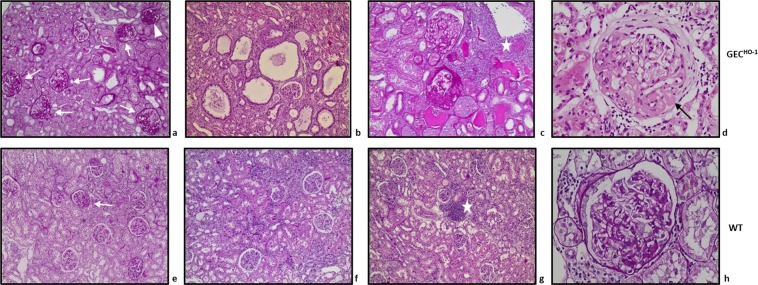
Glomerular and non-glomerular injury in aging GEC^HO-1^ rats. Glomerular and tubulointerstitial lesions in 12-month old rats with GEC-targeted HO-1 overexpression (GEC^HO-1^, **a,b,c,d**) and age-matched WT control rats (**e,f,g,h**). In glomeruli of GEC^HO-1^ rats, focal or global segmental sclerosing lesions were more extensive (**a** vs **e**, arrows: segmental sclerosis; arrowhead: global sclerosis, PAS stain x100), as were cystic dilatation of tubular lumens (**b** vs **f**, HE stain x100), interstitial inflammatory infiltrates [stars in c vs g, PAS stain x200 (**c**) and HE stain x100 (**g**)], presence of proteinaceous casts (**c** vs **g**), and thickening of the Bowman’s capsule [**d** vs **h**, HE stain x400 (**d**); PAS stain x400 (**h**)]. Hyalinosis lesions were also present in sclerosed glomeruli of GEC^HO-1^ rats (**d**, arrow). HE: hematoxylin/eosin. PAS: periodic acid Schiff.

Prominent renal lesions in 12-month GEC^HO-1^ rats consisted of focal segmental glomerulosclerosis (FSGS) or focal global glomerulosclerosis (FGGS) (Fig. 2a), cystic dilatation of tubular lumens (Fig. 2b), proteinaceous casts (Fig. 2c), thickening of Bowman’s capsule and hyaline deposits in sclerosed glomeruli (Fig. 2d). Extent of glomerular lesions (expressed as percent of glomeruli with a specific lesion) is shown in Table 2. All lesions in GEC^HO-1^ rats did not reach statistical significance compared to those in age matched WT controls until 12 months of age. There was a statistically significant increase in FSGS, interstitial inflammation, presence of cystically dilated tubules and tubular atrophy in 12-month old GEC^HO-1^ rats compared to age matched WT controls. FSGS was the most prevalent lesion in both GEC^HO-1^ and WT 12-month old rats.

**Table 2:** Glomerular and non-glomerular (tubulointerstitial) lesions in aging WT and GEC^HO-1^ rats.

Animals (Age)						
	2 months		6 months		12 months	
	WT n = 4	GEC^HO-1^ n = 4	WT n = 4	GEC^HO-1^ n = 4	WT n = 4	GEC^HO-1^ n = 4
**Glomerular lesions**						
FGGS	0.0	0.0	0.0	5.0 ± 5.0	0.0	6.2 ± 3.2
FSGS	0.0	1.6 ± 1.1	0.2 ± 0.2	12.5 ± 9.7	6.9 ± 2.4	31.7 ± 9.5^**,##,†^
Thickening of Bowman’s capsule	0.3 ± 0.2	0.4 ± 0.3	1.0 ± 0.7	4.7 ± 3.1	2.1 ± 1.7	9.2 ± 4.9^##^
**Non-glomerular lesions**						
Cystically dilated tubules	0.000	0.250 ± 0.250	0.000	0.500 ± 0.289	0.250 ± 0.250	1.000 ± 0.000^*,##^
Interstitial inflammation	0.000	0.000	0.000	0.750 ± 0.750	0.750 ± 0.479	2.250 ± 0.479^*,##,†^
Interstitial fibrosis	0.000	0.000	0.000	0.500 ± 0.289	0.250 ± 0.250	0.750 ± 0.250^##^
Tubular atrophy	0.000	0.000	0.000	0.000	0.250 ± 0.250	0.750 ± 0.250^*,##,††^

Comparison of extent of glomerular and tubulointerstitial lesions in WT and GEC^HO-1^ rats at 2-, 6-, and 12-months of age (n = 4 at each time point for WT or GEC^HO-1^). In kidney sections of each individual animal at least 50 glomeruli were examined for presence of lesions, as described in Methods, and number of glomeruli with a specific lesion was noted and expressed as % value. The mean % value ± SEM from all 4 WT or GEC^HO-1^ animals was then determined. Interstitial inflammation was graded on a scale of 0 to 4 as described in Methods and expressed as mean ± SEM. Presence or absence of cystically dilated tubules, interstitial fibrosis and tubular atrophy was recorded as 1 or 0 respectively, and expressed as mean ± SEM. *p < 0.05 vs WT 12-month old, **p < 0.01 vs WT 12-month old, ^##^p < 0.01 vs GEC^HO-1^ 2-month old, ^†^p < 0.05 vs GEC^HO-1^ 6-month old, ^††^p < 0.01 vs GEC^HO-1^ 6-month old.

**Figure 3:**
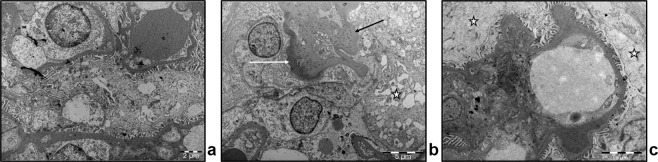
Electron microscopy of 12-month old GEC^HO-1^ rat glomeruli compared to age-matched WT. (**a**) Electron microscope image (magnification: x5600) of glomeruli in a 12-month old WT rat showing well-preserved foot processes and glomerular basement membrane. (**b,c**) Electron microscope images (magnification: x4400) of glomeruli in a 12-month old GEC^HO-1^ rat with segmental glomerular lesion, hyalinosis (white arrow in **b**), wrinkled basal lamina (black arrow in **b**), extensive effacement of foot processes and abundant vacuoles within epithelial cells (star in **b**) and GEC swelling (stars in **c**).

Glomeruli of 12-month old GEC^HO-1^ rats with lesions of glomerular sclerosis were further examined by electron microscopy. As shown in Fig. 3b,c, there were prominent changes in visceral glomerular epithelial cells (podocytes) including edematous cell bodies, areas of foot process effacement, thickening and wrinkling of the glomerular basement membrane and increased number of vesicles reflecting endocytotic activity compared to age matched WT control (Fig. 3a).

### HO-1 expression and hemosiderin deposition

**Figure 4:**
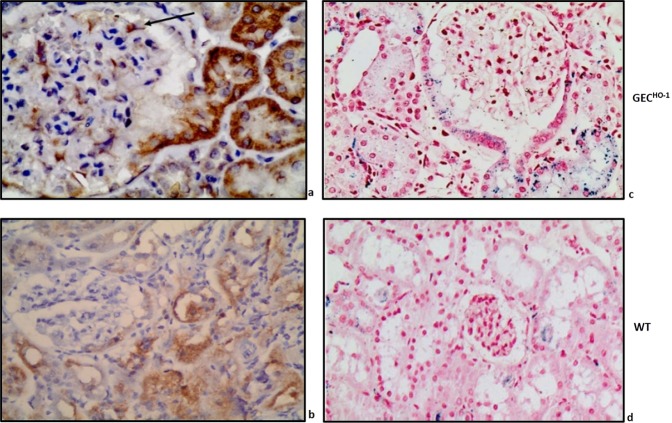
ΗΟ-1 expression and hemosiderin deposition in aging GEC^HO-1^ rat kidney tissue. (**a**) prominent HO-1 protein immunolocalization in epithelial cells of the proximal tubule arising from the urinary pole of the glomerulus and in epithelial cells of surrounding tubules (x600). HO-1 is also expressed in podocytes (black arrow), as expected from targeted HO-1 overexpression achieved in these cells. (**b**) HO-1 protein expression in an albuminuric WT rat (less prominent and extensive in tubules) (x400). Hemosiderin staining (Prussian blue, x400) in kidney sections of 12-month old GEC^HO-1^ (**c**) and WT rat (**d**).

Figure 4 shows immunolocalization of HO-1 in an albuminuric 12-month old GEC^HO-1^ rat. Prominent HO-1 protein expression was present in epithelial cells of the proximal tubule arising from the urinary pole of the glomerulus shown (Fig. 4a) and in epithelial cells of surrounding tubules. In the former, epithelial cells were also positive for hemosiderin (Fig. 4c). HO-1 protein was also expressed in glomerular podocytes (Fig. 4a), as expected from targeted HO-1 overexpression in these cells achieved using the hyperactive SB transposon system. In an albuminuric WT rat (no podocyte-targeted HO-1 overexpression), HO-1 protein expression in tubules was less prominent and extensive while there was no expression in podocytes (Fig. 4b). Hemosiderin deposits in tubules of albuminuric WT rats were sparse (Fig. 4d).

**Figure 5:**
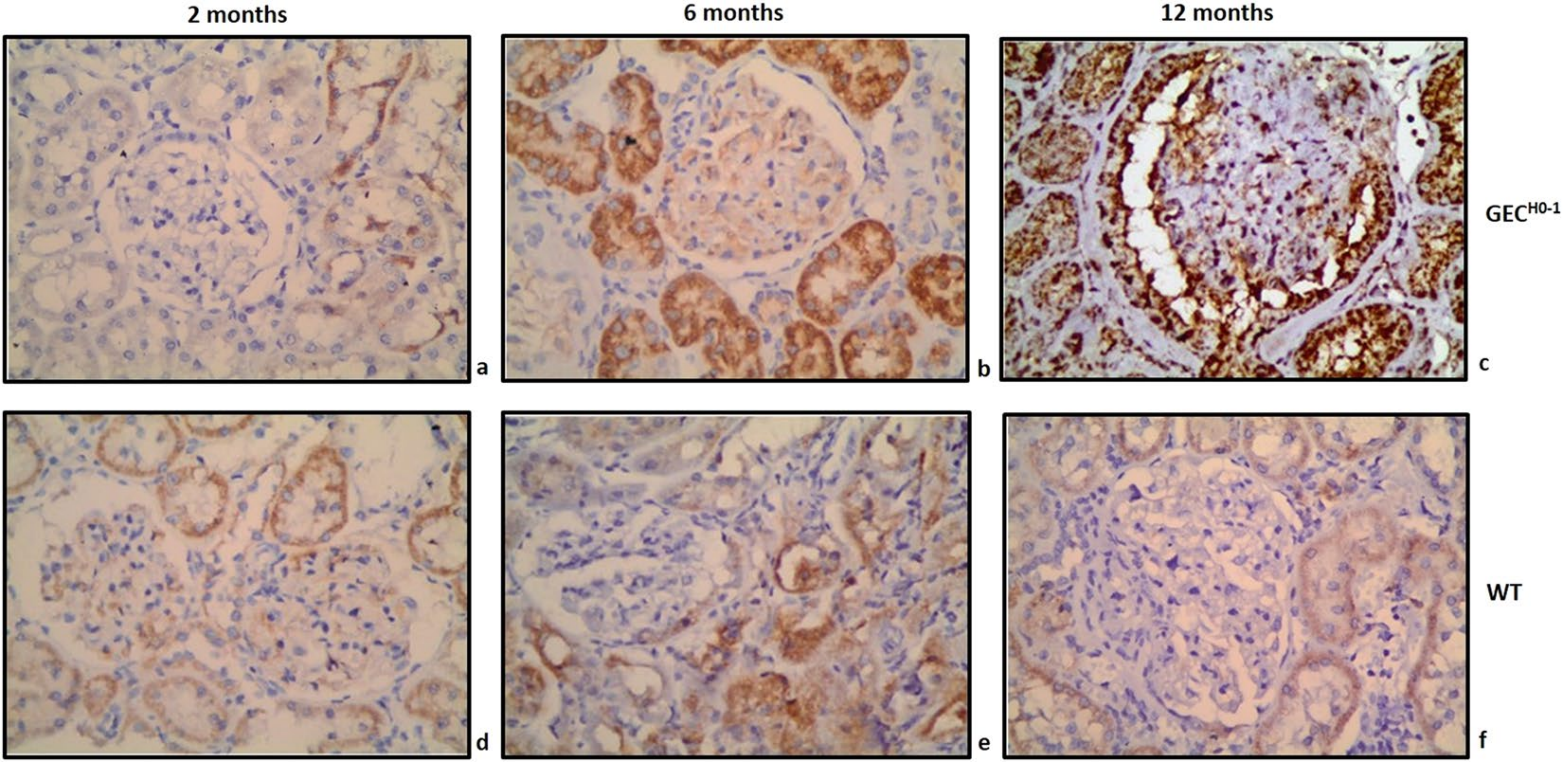
Changes in HO-1 expression in aging GEC^HO-1^ and WT rats. Immunolocalization of HO-1 protein HO-1 expression in cortical sections of 2-, 6-, and 12-month old GEC^HO-1^ rats and in age-matched WT controls, magnification x400. Age-dependent increase in HO-1 expression in GEC^HO-1^ rats (**a,b,c**) compared to WT controls (**d,e,f**).

**Figure 6:**
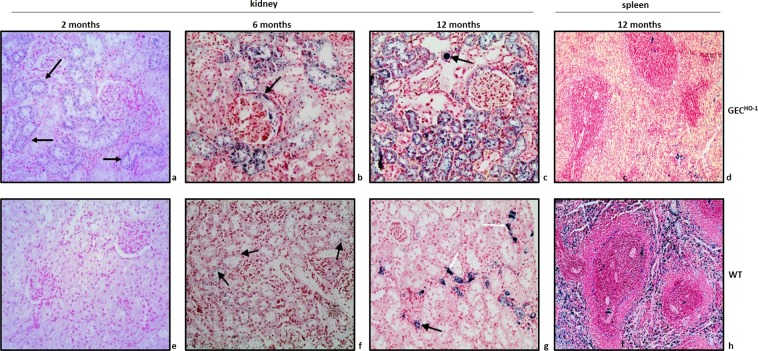
Hemosiderin deposition in kidney and spleen sections of aging GEC^HO-1^ and WT rats. Hemosiderin deposition (Prussian blue stain x200) in renal cortical sections of 2-, 6-, and 12-month old GEC^HO-1^ rats and age-matched WT controls as well as in spleen sections of 12-month old GEC^HO-1^ rats and age-matched WT controls. Fine hemosiderin granules in cytoplasm of tubular epithelial cells became apparent as early as 2 months in GEC^HO-1^ rats (**a**). Coarse granules of hemosiderin deposition in tubular epithelial cells and in lumen of distal tubules (c, black arrow) of 6- and 12-month old GEC^HO-1^ rats (**b**,**c**). Deposition most prominent at 12 months. No detectable hemosiderin in cortical section of 2-month old WT rat (**e**). Detectable but sparsely distributed hemosiderin in cortical sections of 6- and 12-month old WT rats (**f**,**g**, arrows). Markedly reduced hemosiderin deposition in spleen sections of 12-month-old GEC^HO-1^ rats (**d**) compared to age-matched WT controls (**h**).

Immunolocalization of HO-1 protein in cortical sections of 2, 6, and 12-month old GEC^HO-1^ rats and in age-matched WT controls is shown in Fig. 5. Hemosiderin deposition at same time points is shown in Fig. 6. There was an age-dependent increase in both HO-1 expression and hemosiderin deposition particularly in GEC^HO-1^ rats in which hemoglobinuria was also present (Table 1).

### Depletion of iron stores in GEC^HO-1^ rats

As hemosiderin is composed of ferritin aggregates and ferritin is a major iron storage complex most commonly found in macrophages, the observation that GEC^HO-1^ rats developed hemoglobinuria with marked hemosiderin deposition in renal tubules raised the question of whether these animals became depleted of iron stores. To address this question, spleen sections (an abundant source of various macrophage types) of GEC^HO-1^ rats with hemosiderin deposition in renal tubules were also stained for Prussian blue. As shown in Fig. 6, there was marked iron depletion (assessed as hemosiderin deposition) in spleen sections of 12-month old GEC^HO-1^ rats (Fig. 6d) compared to spleen sections of WT controls (Fig. 6h).

### Assessment of injury

**Figure 7:**
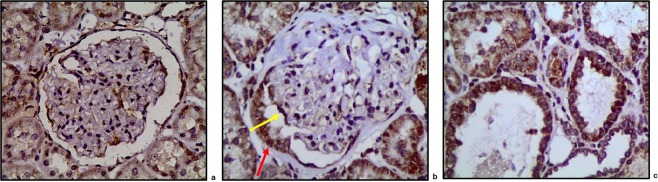
Presence of oxidative stress in aging GEC^HO-1^ rats. Immunolocalization of 8-Ohdg (**a** marker of oxidative RNA and DNA damage by reactive oxygen and nitrogen species) in GEC^HO-1^ 12-month old rat. Prominent 8-OHdG immunolocalization detected in podocytes (**b**, arrow) and in tubular epithelial cells of an albuminuric GEC^HO-1^ rat (**b**,**c**) compared to a 12-month old WT (**a**), magnification x400.

**Figure 8:**
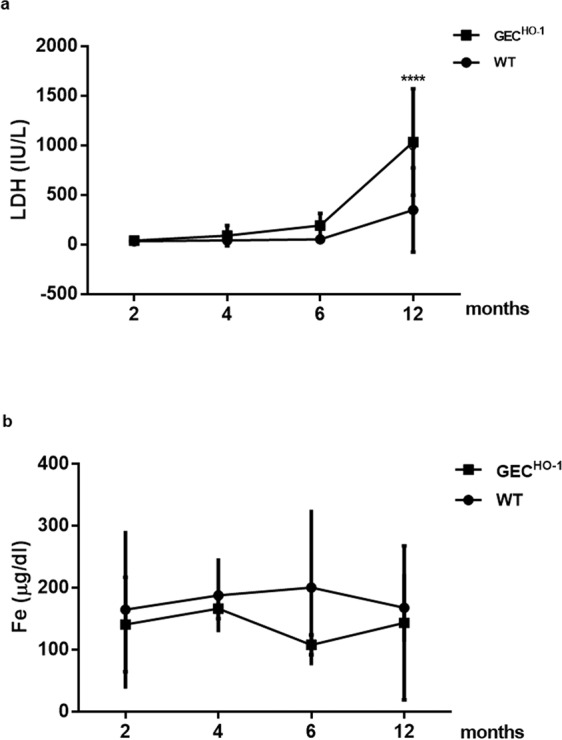
Changes in serum lactate dehydrogenase (LDH) and iron (Fe) levels in GEC^HO-1^ and WT rats. Serum LDH (IU/L) and Fe (μg/dL) in 2-, 4-, 6-, and 12-month old WT and GEC^HO-1^ rats. Values are expressed as mean ± SEM. (**a**): Significant increase in LDH at 12-month old GEC^HO-1^ rats compared to WT controls. (**b**): No significant changes in serum iron. ****p < 0.0001 12 month old GEC^HO-1^ vs 12 month old WT.

As hemoglobinuria and consequent iron deposition in tubular epithelial cells can cause oxidative injury, we examined expression of 8-hydroxydeoxyguanosine (8-OHdG), a marker of oxidative damage to RNA and DNA by reactive oxygen and nitrogen species. There was prominent 8-OHdG immunolocalization primarily in podocytes and tubular epithelial cells of albuminuric 12-month old GEC^HO-1^ rats (Fig. 7b,c) compared to WT controls (Fig. 7a), which showed sparse 8-OHdG staining. To determine whether the extent of injury was of sufficient magnitude to increase serum lactate dehydrogenase (LDH), we measured serum LDH levels in 2, 6, and 12-month old GEC^HO-1^ rats and age-matched WT controls. We also measured serum iron levels to assess whether hemoglobinuria in GEC^HO-1^ rats was associated with iron depletion. As shown in Fig. 8, serum LDH increased at 12 months in both GEC^HO-1^ and WT control rats and was significantly higher in the former. There were no significant changes in serum iron levels.

## Discussion

The present studies demonstrate that podocyte-targeted HO-1 overexpression exacerbates albuminuria and extent of glomerular and tubulointerstitial lesions in ageing Sprague-Dawley rats. This outcome was unexpected in view of previous observations that pharmacological activation of Nuclear factor-erythroid 2 related factor 2 (Nrf2), a transcription factor that upregulates HO-1, was shown to ameliorate aging-related progressive renal injury^20^, and that systemic treatment with the natural HO-1 substrate/inducer, hemin, preserves podocyte structural/functional integrity and ameliorates extent of glomerular and tubulointerstitial lesions under conditions of oxidative stress^17^. However, attenuation of renal injury observed in these studies occurred without a clear demonstration of HO-1 induction in podocytes. Moreover, podocyte HO-1 induction does not occur in response to exogenous administration of HO-1 inducers including heme^18^ or in forms of glomerular injury associated with generation of potent HO-1 inducers within glomeruli^19^. Therefore, the role of HO-1 induction in podocytes in attenuating renal injury described in these studies could not be determined. Podocyte-targeted HO-1 induction, achieved in the present study, and assessment of structural/functional outcomes in the ageing rat kidney allowed for a definitive determination of this role.

A number of mechanisms could account for detrimental effects observed in rats with podocyte-specific HO-1 induction. Detrimental rather than beneficial effects of HO-1 induction were previously demonstrated in other systems and depend on the cell type in which induction occured^21^. Although heme degradation following HO-1 induction generates biliverdin/bilirubin and induces ferritin thereby exerting an overall antioxidant effect, compromised cell membrane integrity in cells over expressing HO-1 also occurs^22^, while ferrous iron (Fe^++^) derived from heme degradation can reverse cytoprotective effects of HO-1 when high expression levels are achieved^23^. However, the reversal of HO-1 cytoprotective effect by ferrous iron was shown in cultured hamster fibroblast only^23^ and may not apply to other highly specialized cell types such as the podocyte. A more likely mechanism in ageing GEC^HO-1^ animals could involve podocyte hemoglobin uptake since sustained hemoglobinuria developed in this model. Cultured human podocytes or podocytes isolated from murine glomeruli were shown to bind and endocytose hemoglobin (Hb) through the megalin-cubilin receptor system, thus resulting in increased intracellular Hb catabolism, oxidative stress, activation of apoptosis pathways, and abnormal morphology including decreased expression of the slit diaphragm proteins nephrin and synaptopodin. Moreover, mice with experimentally induced hemolysis develop proteinuria and demonstrate podocyte injury, characterized by foot process effacement, decreased synaptopodin and nephrin expression, and podocyte apoptosis^24^.

The “forced” human HO-1 over expression (an endoplasmic reticulum-anchored protein) achieved in podocytes of GEC^HO-1^ rats may also contribute to injury. A similar outcome was previously described in a transgenic rat model of podocyte retention of a non-podocyte protein within the endoplasmic reticulum (ER). It was demonstrated that accumulation of the protein in the podocyte ER results in ER stress and podocyte injury^25^. An alternative mechanism of podocyte injury consequent to HO-1 overexpression may involve increased heme degradation resulting in depletion of free heme. Heme depletion was shown to phosphorylate the heme-regulated eukaryotic initiation factor-2a (eIF2α kinase), which has been termed heme-regulated inhibitor and heme-controlled repressor. eIF2α phosphorylation has a strong inhibitory effect on initiation of protein synthesis^26^. In glomeruli of proteinuric rats, eIF2α phosphorylation is enhanced while eIF2alpha phosphorylation in cultured GECs reduces protein synthesis^27^.

Histological changes of rat kidney senescence are known to appear at approximately 6 months^2^. In 12-month-old GEC^HO-1^ rats albuminuria as well as extent of segmental glomerulosclerosis increased (Figs. 1 and 2) with a highly significant increase in FSGS and FGGS lesions. Such lesions are frequently encountered in the ageing kidney both in humans and in animal models^1^ while the number of podocytes in glomeruli having these lesions decreases^6^. The present study corroborates previous observations^28^ that, despite worsening of albuminuria and progression of ageing-related lesions in WT rats older than 6 months, serum urea and creatinine levels were not higher than those in younger WT animals. In contrast, serum creatinine was significantly higher in 12-month old GEC^HO-1^ rats, indicating that injury as a consequence of GEC-targeted HO-1 overexpression had an adverse effect on glomerular filtration.

Renal injury was apparently oxidative in nature as documented by immunolocalization of 8-hydroxydeoxyguanosine (a marker of oxidative damage to RNA and DNA by reactive oxygen and nitrogen species) in podocytes (Fig. 7b) and in tubular epithelial cells (Fig. 7c), and the extent of injury was sufficient to increase serum LDH (Fig. 8). Causative mechanisms of oxidative injury in GEC^HO-1^ rats include exposure of tubular epithelial cells to hemoglobin/heme and deposition of iron as hemoglobinuria (presence of blood but not erythrocytes in urine) was detected in GEC^HO-1^ but not WT rats (Table 1) and was sufficient in magnitude to cause depletion of iron stores in the former as shown in Fig. 6 (panel d vs h). A plausible mechanism for hemoglobinuria is injury/lysis of erythrocyte membrane and release of hemoglobin during erythrocyte passage through damaged glomeruli. Erythrocyte lysis could be a contributory factor for increased serum LDH observed. Hemoglobin endocytosis by proximal tubular cells^29^ and subsequent release of heme can induce HO-1 and this could explain the marked increase in HO-1 expression observed in GEC^HO-1^ rat tubules (Figs. 4a and 5). The ensuing generation of heme-derived iron can induce tubular cell ferritin synthesis thereby further contributing to hemosiderin (a ferritin complex) formation, as shown in Figs. 4c and 6. Albuminuria can further contribute to iron overload since iron bound to filtered serum transferrin or ferritin is presented to tubular cells via the tubular lumen where the acid milieu favors its release^30^.

In summary, the present study demonstrates that podocyte-targeted HO-1 overexpression exacerbates severity of ageing-related renal pathology, with statistically significant increase in FSGS, FGGS and tubulointerstitial lesions. The mechanism(s) remain to be explored and may involve, (a) depletion of podocyte free heme, (b) reversal of cytoprotective effects of HO-1 due to increased release of Fe^++^ from heme as a result of high HO-1 protein levels in podocytes, and (c) increased iron deposition in tubules.

## Materials and Methods

### Animals

Animals were reared in accordance to the European Union Directive for care and use of laboratory animals and all procedures were approved by the Hellenic Veterinary Administration and the ethical committee of ‘Evangelismos’ Hospital. Rats with visceral glomerular epithelial cell (podocyte)-targeted HO-1 overexpression (GEC^HO-1^) rats were generated as we described previously^31^ using the hyperactive SB transposon system and a colony was established^31^. Briefly, an SB transposon vector (SB-hHO1) was constructed harboring a human (h) HO-1 sequence under control of a murine nephrin promoter previously used to achieve podocyte-targeted hHO-1 expression in the mouse^32^. The SB transposon system methodology, as reported by Katter *et al*.^33^, was then applied. The vector was mixed with SB100 transposase mRNA and injected into the pronucleus of newly fertilized Sprague-Dawley (SD) embryos before transferring to pseudopregnant females. Insertions were mapped by rat genome digestion with Bfa1 and linker-mediated polymerase chain reaction (PCR) amplification in order to obtain a sequence tag that could be “blasted” thereby determining: a) the specific chromosome location of the transposon, and b) whether the transposon was incorporated in an exon or intron. Sequences obtained from ligation-mediated PCR (LM-PCR) amplification were subjected to rat genome analysis by comparison to the Baylor 3.4/rn4 reference sequence at http://genome.ucsc.edu/ to map the precise transgene integration sites.

All rats were genotyped following rat tissue (tail or ear) DNA extraction using the DNeasy Blood and Tissue kit (Qiagen, Manchester, UK) and the following PCR amplification primer pairs: SB1F 5′-GAG GGA AGA GAG AAG GGC GAGT-3′ and SB1R 5′ – CCT TGT TGC GCT CAA TCT CCT-3′ and SB2F 5′-CGA CAG CAT GCC CCA GGA TT-3′ and SB2R 5′-CTC TGG GAG TCT CCA CGG GG-3′. Following an initial denaturation step (95 °C for 5 min), samples were subjected to 30 cycles of denaturation at 95 °C (1 min), annealing (60 °C for 1 min) and extension (72 °C for 1 min). A final extension step (72 °C for 1 min) was performed.

### Study design

A total of sixteen GEC^HO-1^ rats from our established colony were used in the study (n = 4, for each time point). The same number of WT rats served as controls. All rats were maintained in cages with access to water during the urine collection. Only male rats were used in the study as it has been shown that male ageing rats were shown to be more susceptible in developing both glomerular and tubulointerstitial lesions^34^. The effect of podocyte-targeted HO-1 overexpression on aging-related lesions was examined in 2, 4, 6 and 12-month old rats. At these time points animals were placed in individual metabolic cages to obtain urine samples. They were subsequently anesthetized, blood samples were obtained and nephrectomies were performed.

### Renal function and hematology studies

Urine albumin and creatinine concentrations ware measured using commercially available ELISA kits (Nephrat kit, Exocell, PA, USA) and albuminuria was expressed as Ualb/Ucr ratio. Urine samples were also examined for presence of blood (Hemoglobin, Hb) using cellulose strips impregnated with hydrogen peroxide and a chromagen (COMBI SCREEN® SYS, Analyticon Biotechnologies AG, Germany). The method is based on the peroxidase properties of hemoglobin to catalyze the reaction between hydrogen peroxide and the chromagen. Results were expressed semiquantitatively based on a 0 to +++ scale. Serum urea, creatinine, LDH and iron were measured in an automated sampler (Medilyzer BT, Medicon, Athens, Greece).

### Histopathology and immunohistochemistry

Rat kidney and spleen tissues were fixed in formalin-buffered saline for 24 h, followed by dehydration with graded ethanols (80–90%), embedding in paraffin and sectioning (0.5-μm-thick sections). Serial sections were applied to Poly-L-Lysine coated slides and left at 55 °C to remove excess paraffin. Routine procedures were subsequently followed for staining kidney sections with hematoxylin/eosin (HE), periodic acid Schiff (PAS), and Prussian blue (detection of hemosiderin, a ferritin complex). Spleen sections were stained with hematoxylin/eosin (HE) and Prussian blue.

Renal tissues from GEC^HO-1^ and age-matched WT rats were examined by light microscopy. Extent of glomerular lesions was assessed in each individual animal at 2, 4, 6 and 12 months of age. For each time point, 4 GEC^HO-1^ rats and 4 age-matched WT controls were studied. In kidney sections of each individual animal glomerular lesions were semi-quantified^35^ while genotype of the animal was blinded to eliminate potential experimental bias. Briefly, at least 50 glomeruli were examined for presence of lesions and number of glomeruli with a specific lesion in each animal was noted and expressed as % value. The mean % value ± SEM from all 4 WT or GEC^HO-1^ animals was then determined. Tubulointerstitial lesions were graded using a method previously described in a rat model of tubulointerstitial nephritis^36^. Glomerular lesions assessed included: FSGS, FGGS, increase in glomerular matrix, segmental collapse and obliteration of capillary lumina and accumulation of hyaline, synechial attachments of the glomerular tuft to Bowman’s capsule, and thickening of Bowman’s capsule^37^. FSGS was defined as scarring occupying a glomerular segment^37^. FGGS was defined scarring occupying the entire glomerular tuft^35^.

Tubules and the interstitial space were also examined and presence or absence of tubular atrophy, cystically dilated tubules and interstitial fibrosis was recorded. Interstitial inflammation was graded on a scale of 0 to 4 (0 = normal; 0.5 = small focal areas of inflammatory infiltration; 1 = inflammatory infiltration in less than 10% of the cortex; 2 = inflammatory infiltration in up to 25% of the cortex; 3 = inflammatory infiltration in 50 to 75% of the cortex; and 4 = extensive damage involving more than 75% of the cortex), as previously described^36^. Presence or absence of cystically dilated tubules, interstitial fibrosis and tubular atrophy was recorded as 1 or 0, respectively.

For immunolocalization of HO-1 in kidney sections, an anti-HO-1 monoclonal antibody (cat no: ADI-SPA-895, Enzo Life Sciences, NY, USA) was used at a 1:100 dilution. To assess presence of oxidative stress, immunohistochemical staining for 8-hydroxydeoxyguanosine (8-OHdg, a byproduct of oxidative DNA damage) was performed using a monoclonal, anti-8-OHdg antibody (cat no: sc66036, Santa Cruz) at a dilution of 1:50. Immunohistochemistry was carried out using the Ultra Vision Quanto Detection System HRP DAB (Thermo Scientific) according to the manufacturer’s instructions.

### Electron microscopy

Kidney tissue was minced into 1-mm pieces and fixed in 2.5% glutaraldehyde in PBS followed by post fixation in 1% osmium tetroxide in PBS for 1 h. Subsequently, sections were dehydrated in graded ethanol dilutions and embedded in a mixture of Araldite CY212, Epon 812, DDSA, dibutyl ester and DNP 30 hardener. Sections were cut at 90-nm thickness using a Leica Ultracut UCT (Leica Microsystems, Vienna, Austria) and stained with uranyl acetate and lead citrate. Images were captured at 80 Kv using a Fei electron microscope (Hillsboro, OR, USA) equipped with a CCD camera.

### Statistical analysis

Values were expressed as mean ± SEM (standard error of the mean). The GraphPad Prism program was used for statistical analyses (*GraphPad Software Inc*). Analyses were performed with analysis of variance (two-way ANOVA) for more than two group comparisons. When significant, post hoc analysis was performed, with the Fisher’s least significant difference (LSD) test. A p value < 0.05 was chosen as statistically significant.

## Data Availability

All data generated or analyzed during this study are included in this published article.
